# Intradermal needle-free injection prevents African Swine Fever transmission, while intramuscular needle injection does not

**DOI:** 10.1038/s41598-023-31199-2

**Published:** 2023-03-21

**Authors:** Muhammad Salman, Hongyao Lin, Roypim Suntisukwattana, Parin Watcharavongtip, Patumporn Jermsutjarit, Angkana Tantituvanont, Dachrit Nilubol

**Affiliations:** 1 Swine Viral Evolution and Vaccine Development Research Unit, Department of Veterinary Microbiology, Faculty of Veterinary Science, Chulalongkorn University, Henry Dunant Road, Pathumwan, Bangkok, 10330 Thailand; 2 MSD Animal Health Innovation Pte Ltd, Singapore, 718847 Singapore; 3 Department of Pharmaceutic and Industrial Pharmacies, Faculty of Pharmaceutical Sciences, Chulalongkorn University, Bangkok, 10330 Thailand

**Keywords:** Microbiology, Diseases

## Abstract

Shared needles are a possible iatrogenic and hematogenous inanimate vector of African Swine Fever virus (ASFV) in farm conditions. To evaluate that possible transmission, sixty, 4-week-old pigs were procured from an ASF free herd free. Upon arrival, pigs were randomly divided into two sets. Set 1 served as seeder pigs, and were randomly allocated to 4 groups. The other pigs were divided into 8 groups, and served as sentinels. Seeder pigs were oronasally challenged with ASFV at high (10^8^ copy numbers/mL), moderate (10^6^ copy numbers/mL) or low (10^1^ copy numbers/mL) challenge titer, except a subgroup that remained unchallenged (negative control). At 7 days post challenge (peak viremia), all four seeder groups were intradermally and intramuscularly (IM) injected with a vaccine adjuvant (Diluvac Forte, MSD Animal Health, The Netherlands) using a needle-free device (IDAL 3G, MSD Animal Health, The Netherlands) and conventional needles, respectively. The same needle or needle-free device was then used to inject the same volume of adjuvant into set 2 (n = 48) pigs. All pigs were observed for clinical disease daily and assayed for the presence of ASFV DNA by quantitative PCR. All seeder groups developed viremia (except the control pigs). ASFV viremia was detected in all sentinel groups injected via the intramuscular route. Transmission rate from the IM route via conventional needles was positively correlated with virus titer in blood circulation of seeders. Sentinels intramuscularly exposed to needles from high titer challenged seeders displayed more severe and acute clinical disease compared to that of exposed to low titer challenged seeders. No viremia nor clinical signs were observed in the sentinel groups injected via the intradermal route. This study confirmed the hematogenous transmission of ASFV between pigs through needle-sharing.

## Introduction

African swine fever (ASF) is a lethal disease of pigs emerging globally and is gaining momentum in Asia since its introduction to China in August 2018^1^, followed by several countries in Southeast Asia^2–6^. ASF virus (ASFV), a large double-stranded DNA virus and a member of the *Asfarviridae* family, is the causative agent. Its large genome contains 151–160 open reading frames encoding more than 170 proteins and 24 genotypes including genotypes I–XXIV have been recognized^7^. Genotype II is currently the dominant genotype circulating in several European and Asian countries^1^. Although the main risk factors for the transmission of the ASF virus (ASFV) between herds are live animal transportation and poor compliance with biosecurity measures^8^, the abundance of small farms and the recent emergence of diversified ASFV strains including less virulent ones^9^ represent new threats to the global swine industry. Currently, there are no effective and safe ASFV vaccines. ASF control relies mainly on strict biosecurity and early detection to eliminate infected pigs. Vaccination against common swine diseases including Porcine Reproductive and Respiratory Syndrome, Porcine circovirus associated diseases, classical swine fever and *Actinobacillus pleuropneumoniae* is routinely performed on commercial swine farms. Vaccination is routinely practiced using conventional needles, mostly via the intramuscular (IM) route, although vaccination through the intradermal (ID) route is efficacious against major swine pathogens and commercially available^10,11^. The IM procedure may transmit viruses and bacteria between animals, especially for diseases with a delayed onset of clinical signs or with subclinical infection. Pigs infected with low virulence strains may demonstrate no symptoms of ASF but serve as harbors of infection within the herd, possibly through hematogenous transmission by injection, since disposable injection needles are often used for several animals and good vaccination practices are variably implemented^12^.

It has previously been demonstrated that disposable conventional needles are able to iatrogenically transmit that virus between pigs, while the use of an ID vaccination device was not able to^13^. An additional observation was intramuscular vaccination using conventional needles induced a significantly higher level of IL-10 compared to intradermal vaccination using needle-free device^14^. The objectives of the present study were to assess whether these results could be replicated using ASFV^15^, and to determine whether or not the virus load in pigs is associated with transmission. To do so, and because ASF is a regulated disease, the trial was performed in a highly biosecure experimental farm, where viremic (seeder) pigs were produced by direct inoculation with the ASFV and where ASF-naïve pigs (sentinels) were exposed through injection by either the IM (needle) or the ID route (design ID vaccination device). Our hypotheses were that: (i) our experimental ASFV transmission to seeder pigs would be efficient, even with low-titer inoculum, (ii) a needle that had just been used for IM injection of a viremic (seeder) pig would transmit ASFV to the next pig injected (sentinel) and (iii) Viremia levels in pigs inoculated with a low-titer inoculum would be too low to allow transmission in our experimental protocol.

## Materials and methods

### Infectious inoculums

The inoculum used in the study originated from infected pigs during a field outbreak in Thailand in 2022. Spleens from clinically affected pigs were weighed and mixed with Dulbecco’s minimum essential medium (DMEM, Gibco, MA, USA) supplemented with 5 × antibiotics (100 × Antibiotics-antimycotics, Gibco, MA, USA) to prepare a 10% weight by volume (w/v) suspension. The tissue was then homogenized and centrifuged at 4000*g* for 10 min. Homogenate was filtered through 0.2 μm pore size filter, treated with 5 × antibiotics overnight at 4 °C, and kept at − 80 °C until used. To prepare the inoculums, homogenate suspension was serially tenfold diluted with DMEM and quantified for the ASFV genomic copy numbers per mL using real time-qPCR as described below.

### Study design

The study is reported in accordance with the ARRIVE guidelines (https://arriveguidelines.org). The experiments took place on an experimental farm with a high biosecurity level.

Sixty, 4-week-old male castrated pigs were procured from a conventional Thai herd free of ASFV, Classical Swine Fever and PRRS, where all pigs are vaccinated at weaning (3 weeks of age) against Porcine Circovirus type 2 and enzootic pneumonia. Upon arrival at the experimental facility, pigs were individually identified (ear tagged) and then randomly allocated based on stratification by weight into two sets (Table 1). Set 1 pigs (n = 12) were to be used as seeders. They were randomly allocated into 4 subgroups (3 pigs per subgroup), to be left uninoculated (control) or to be inoculated with high-, moderate- or low ASFV titers (ASF-H, ASF-M, ASF-L subgroups, respectively). Set 2 pigs (n = 48) were to be used as sentinels. They were age-matched with set 1 pigs, and then randomly allocated into 8 subgroups of 6 pigs each, to be injected with a deliberately non-disinfected device used minutes before in viremic seeder pigs. During the duration of the trial, each group was kept in a separate room during the entire trial, with free and ad libitum access to feed and water. Operators entering rooms wore personal protective equipment and disinfected their equipment upon exiting each room.

**Table 1:** Experimental design of the ASFV transmission study.

Seeders		ASFV Challenge	Sentinels		Details	Dosage of solvent, route and injection device
Seeder pigs	No. of seeder pigs		Sentinel pigs	No. of sentinel pigs		
ASF-H	3	High dose (10^8^ copy number/mL)	Sentinel H-ID	6	Needle-less injection	0.2 mL of Diluvac^®^ Forte, Intradermal using IDAL^®^ 3G (MSD Animal Health, The Netherlands)
			Sentinel H-IM	6	Needle injection	2 mL of Diluvac^®^ Forte, Intramuscular using conventional needles (no. 18, 1″)
ASF-M	3	Medium dose (10^6^ copy number/mL)	Sentinel M-ID	6	Needle-less injection	0.2 mL of Diluvac^®^ Forte, Intradermal using IDAL^®^ 3G (MSD Animal Health, The Netherlands)
			Sentinel M-IM	6	Needle injection	2 mL of Diluvac^®^ Forte, Intramuscular using conventional needles (no. 18, 1″)
ASF-L	3	Low dose (10^1^ copy number/mL)	Sentinel L-ID	6	Needle-less injection	0.2 mL of Diluvac^®^ Forte, Intradermal using IDAL^®^ 3G (MSD Animal Health, The Netherlands)
			Sentinel L-IM	6	Needle injection	2 mL of Diluvac^®^ Forte, Intramuscular using conventional needles (no. 18, 1″)
Control	3	No ASF challenge	Control-ID	6	Needle-less injection	0.2 mL of Diluvac^®^ Forte, Intradermal using IDAL^®^ 3G (MSD Animal Health, The Netherlands)
			Control-IM	6	Needle injection	2 mL of Diluvac^®^ Forte, Intramuscular using conventional needles (no. 18, 1″)

On day 0, seeder pigs in ASF-H, ASF-M, ASF-L groups were oronasally challenged with 4 mL of an inoculum (2 mL via the oral route and 1 mL per nostril) titering either 10^8^, 10^6^, or 10^1^ copy numbers/mL, respectively. The control subgroup (Set 1) was not inoculated.

At 7 days post challenge (DPC), all pigs in the seeder pig subgroups were first intradermally (ID) injected on the right side of the neck with 0.2 mL of Diluvac^®^ Forte using a needle-free device (IDAL^®^ 3G, MSD Animal Health, The Netherlands), and then intramuscularly (IM) injected on the left side of the neck with 2 mL of Diluvac^®^ Forte, using a conventional needle (18-gauge, 1 inch). Operators took all precautions to ensure the avoidance of cross contamination of devices. Subsequently, the same—deliberately non-disinfected—devices were used to inject sentinel pigs (2 sentinel pigs per device used on a single seeder pig) with the same volume of Diluvac^®^ Forte (0.2 mL for ID and 2 mL for IM injections). Again, all precautions were taken to ensure that no surface was touched with the injection devices except the skin of the sentinel pigs. Diluvac^®^ Forte is a proprietary adjuvant for vaccines manufactured by MSD Animal Health, with a recognized safety in pigs. It was chosen as a proxy to any swine vaccination product administered parenterally.

The main outcome of the study was the viremic status of the pigs, evaluated through Ct values obtained by qPCR, as a proxy to viral load. Secondary outcomes were the detection of viremia in the first pig within a sentinel subgroup, reflecting successful transmission of the ASFV, and mortality levels within each subgroup, reflecting successful injection-borne infection and/or transmission dynamics within the room.

### Clinical evaluation

Following challenge (seeder pigs) or injection (sentinel pigs), rectal temperature, clinical abnormalities, and death were recorded daily for individual pigs. Clinical scoring was used to characterize the progression of ASF, as described elsewhere^16^. Clinical evaluation was performed on all animals until the endpoint (behavior score of 4/4 and/or neurologic score of 2/2), when they were humanely euthanized. Clinical scoring and rectal temperature were performed by the same personnel at the same time of day over the whole duration of the study. All surviving pigs at the end of the study were humanely euthanized using overdose pentobarbital injectable solution. All euthanized pigs underwent necropsy in a dedicated secure facility on the experimental farm.

### Quantification of ASFV genomic copy numbers

Blood samples were collected from all pigs on 0, 7, 14, 21, 28 and 35 DPC, and assayed for the presence of ASFV DNA by real time PCR. Total DNA was extracted from samples using a commercial DNA extraction kit (NucleoSpin^®^ Tissue, Macherey-Nagel, Duren, Germany) in compliance with the manufacturer’s instructions. The DNA quality was measured using a NanoDrop spectrophotometer (Colibri Spectrophotometer^®^, Titertek Berthold, Pforzheim, Germany). The copy number of ASFV was then quantified using a real time-qPCR (qPCR). Primers specific for the P72 gene of the ASFV were used; forward primer: 5′-CAATAACCTGTTTGTAACCCC TGAAATAC-3′ and reverse primer: 5′-TGCCAATCTCGGTGTTGATGAGGA-3′. Each qPCR reaction contained 0.1 μg of DNA, 0.2 μM of each primer, 2 × qPCRBIO SyGreen Mix (PCR Biosystems, London, UK), and deionized water to yield a 20 μL final volume. The thermal profile for qPCR was 94 °C for 4 min, followed by 40 cycles of 94 °C for 45 s, 56 °C for 30 s, and fluorescence acquisition at 72 °C for 45 s. The reaction was carried out in a QuantStudio^®^3 Real-time PCR machine (Thermo-Fisher Scientific, Waltman, MA, USA). pGEM^®^-T Easy Vector (Promega, WI, USA) containing the inserted P72 gene of ASFV was used to construct a plasmid standard. A standard curve was generated using serial tenfold diluted plasmid standard of 10^0^–10^8^ copies/μL. Copy number of the ASFV DNA was calculated using a standard curve method. A Ct value of > 35 was considered a negative result.

## Results

### Seeder pigs exposed to higher virus titer displayed more severe clinical signs and higher virus load in blood circulation

Negative control pigs did not develop clinical signs over the 35 days of observation post challenge and none of them developed a detectable ASFV viremia (Table 2).

**Table 2:** Number of seeder pigs presenting ASFV viremia detectable by qPCR (Ct < 35), and number of dead pigs, over the 35 days following oronasal/intramuscular inoculation with the ASFV (challenge).

Seeder pigs	Parameters	Days post challenge							
		0	5	7	10	14	21	28	35
ASF-H	No. of pigs with Ct < 35.00	0/3	2/3	3/3	0/2	2/2	1/1	–*	–
	Average Ct value	≥ 35.00	25.50	20.55	17.90	16.90	28.13	–	–
	SD	0.00	7.20	1.65	1.00	0.00	6.22	–	–
	No. of dead pigs/total no	0/3	0/3	0/3	1/3	1/3	2/3	3/3	3/3
ASF-M	No. of pigs with Ct < 35.00	0/3	2/3	3/3	2/3	1/2	1/1	–	–
	Average Ct value	≥ 35.00	32.90	31.87	30.50	20.55	17.00	–	–
	SD	0.00	3.33	0.29	1.93	1.65	0.00	–	–
	No. of dead pigs/total no	0/3	0/3	0/3	0/3	1/3	2/3	3/3	3/3
ASF-L	No. of pigs with Ct < 35.00	0/3	1/3	1/3	2/3	1/3	2/3	1/3	2/2
	Average Ct value	≥ 35.00	> 35.00	> 35.00	> 35.00	> 35.00	> 35.00	> 35.00	22.67
	SD	0.00	1.42	1.09	2.73	0.64	0.49	1.00	1.82
	No. of dead pigs/total no	0/3	0/3	0/3	0/3	0/3	0/3	0/3	1/3
Negative control	No. of pigs with Ct < 35.00	0/3	0/3	0/3	0/3	0/3	0/3	0/3	0/3
	Average Ct value	≥ 35.00	≥ 35.00	≥ 35.00	≥ 35.00	≥ 35.00	≥ 35.00	≥ 35.00	≥ 35.00
	SD	0.00	0.00	0.00	0.00	0.00	0.00	0.00	0.00
	No. of dead pigs/total no	0/3	0/3	0/3	0/3	0/3	0/3	0/3	0/3

*All pigs in the subgroup died.

All pigs in the ASF-H subgroup died before D28 PC, the first one dying on D10 PC after having presented moderate viremia on D7 PC (Ct = 27.70) and no detectable viremia on D5 PC. The other two had higher viremia levels in the last sample preceding death (Ct = 16.90).

On D7 PC when the injection procedures were performed, all seeder pigs in the ASF-H and ASF-M subgroups had a detectable viremia (Ct values ranging from 14.7 to 34.10), while one out of six pigs in the ASF-L subgroup had detectable viremia (Ct = 34.50).

All pigs in the ASF-M subgroup died before D28 PC (the first death occurred between sampling dates D10 and D14; this animal had no detectable viremia before D7 PC). All dead pigs presented a high level of viremia on the last blood sample preceding death (Ct = 16.90). The two pigs with the longest survival time had detectable viremia on all sampling dates from D3 PC onwards (see Table 1supplementary material).

In the ASF-L subgroup, one out of six pigs died, on the last day of the observation period (D35 PC); its Ct values had oscillated around the threshold of 35.00 over the observation period. It was over this threshold on D28 PC. The two remaining pigs in this subgroup, which had no detectable viremia on D28 PC, were strongly positive on D35 PC, but were still asymptomatic at the time of euthanasia.

### Sentinel pigs exposed to devices from the negative control group

None of the pigs in the sentinel group exposed to injection devices (via IM or ID route) that had been used on the negative control pigs developed clinical signs, nor detectable viremia. All these animals survived over the 35-day observation period (Table 3).

**Table 3:** Number of sentinel pigs presenting ASFV viremia (in bold) detectable by qPCR, and number of dead pigs (in bold), over the 35 days following injection with a needle-free (intradermal, ID) or a needle (intramuscular, IM) device previously used in a viremic seeder pig.

Seeder subgroups	Sentinel subgroups	Parameters	Days post injection					
			0	7	14	21	28	35
ASF-H	Sentinel H-ID	No. of pigs with Ct < 35.00	0/6	0/6	0/6	0/6	0/6	0/6
		No. of dead pigs/total no	0/6	0/6	0/6	0/6	0/6	0/6
	Sentinel H-IM	No. of pigs with Ct < 35.00	0/6	**3/5**	**2/2**	–*	–	–
		No. of dead pigs/total no	0/6	**1/6**	**4/6**	**6/6**	**6/6**	**6/6**
ASF-M	Sentinel M-ID	No. of pigs with Ct < 35.00	0/6	0/6	0/6	0/6	0/6	0/6
		No. of dead pigs/total no	0/6	0/6	0/6	0/6	0/6	0/6
	Sentinel M-IM	No. of pigs with Ct < 35.00	0/6	0/6	**3/6**	**1/1**	–	–
		No. of dead pigs/total no	0/6	0/6	**3/6**	**5/6**	**6/6**	**6/6**
ASF-L	Sentinel L-ID	No. of pigs with Ct < 35.00	0/6	0/6	0/6	0/6	0/6	0/6
		No. of dead pigs/total no	0/6	0/6	0/6	0/6	0/6	0/6
	Sentinel L-IM	No. of pigs with Ct < 35.00	0/6	0/6	0/6	0/6	0/6	**1/6**
		No. of dead pigs/total no	0/6	0/6	0/6	0/6	0/6	0/6
Control	Control-ID	No. of pigs with Ct < 35.00	0/6	0/6	0/6	0/6	0/6	0/6
		No. of dead pigs/total no	0/6	0/6	0/6	0/6	0/6	0/6
	Control-IM	No. of pigs with Ct < 35.00	0/6	0/6	0/6	0/6	0/6	0/6
		No. of dead pigs/total no	0/6	0/6	0/6	0/6	0/6	0/6

*All pigs in the subgroup died.

### Sentinel pigs exposed via needle injection

One of the six sentinel pigs that received an IM injection with a needle used after previous injection to a seeder pig from the ASF-L subgroup developed viremia on D35 post-injection (PI). This pig was asymptomatic at the time of euthanasia (termination of the study).

All the six sentinel pigs that received an IM injection with a needle used after previous injection of a seeder pig from the ASF-M subgroup died before D28 PI. Three of them had died before D14 PI, the remaining three having detectable viremia by this date and died over the following days.

All the six sentinel pigs that received an IM injection with a needle used after previous injection of a seeder pig from the ASF-H subgroup died before D21 PI. The first pig death in this group was registered before D 7 PI. By this date, 3 out of the 5 remaining pigs had developed viremia and died or were euthanized over the next week. The 2 surviving pigs at D14 PI were also viremic and died at D 17 PIP.

### Sentinel pigs exposed via ID injection

None of the 18 sentinel pigs that received an ID injection with a needle-free device used after previous injection of a seeder pig from the ASF-L, ASF-M or ASF-H subgroup developed viremia, nor clinical signs, nor died, over the 35-day observation period.

## Discussion

This study successfully transmitted ASFV to seeder pigs, and the virus was successfully transmitted from those viremic pigs to naïve ones by needle injection, even in the context of a challenge with a low-titer inoculum. Conversely, needle-free ID injection prevented such transmission. These results were in line with a comparable demonstration using the PRRS virus, where shared needles were found to transmit the virus to sentinel pigs regardless of the challenge dose in seeder pigs^14^, while needle-free ID injection failed to do so. This work contributes to the limited knowledge of hematogenous transmission of pathogens in pigs via needle injection. So far, this route of contamination has been shown to be inefficient for swine hepatitis E virus^17^, but has proven effective for PCV2^18^, as for PRRSV and for *Arcanobacterium pyogenes* after artificial contamination of the needle^19^.

The inoculation of naïve pigs with inoculums of a low, medium or high viral titer successfully transmitted the ASFV genotype II virus. In the ASF-H subgroup, all seeder pigs developed both clinical signs and detectable viremia on D7 PC. Pigs in the ASF-M subgroup developed a detectable viremia on D7 PC but with a delayed onset of clinical signs (D10 PC, although a single pig developed fever and redness from D7 PC onwards). A single pig developed detectable viremia on D7 PC in the ASF-L subgroup, and no pig presented clinical signs before D28 PC. These observations agree with recent knowledge on the pathogenicity of the genotype II Eurasian strain of ASFV^20^, including sub-acute and temporary clinically silent infection^21^. These aspects are of relevance for ASF awareness and surveillance. They also confirm that low-titer inoculum may prove infectious via the oronasal route; in a recently reported trial, an inoculum of 3 HAD_50_ was enough to induce infection in wild boar^22^.

ASFV transmission to sentinel pigs via contaminated shared needles was successful for all subgroups, including those receiving an injection with the same needle as ASF-L seeder pigs. This latter result agreed with our hypothesis, although there was a prolonged silent clinical infection in this group. The fact that viremia was detected in a single seeder pig on D7 PC at a low level (Ct 34.5, which translates to 7.3 DNA copy numbers per 1 µg of extracted DNA) might be explanatory. The results from the study confirmed that the minimal infective dose of ASFV is extremely low. It relates to recently published experimental data where the infectious dose of ASFV through intramuscular injection was 0.1 HAD_50_/mL inoculum^23^, while the median infectious dose of ASFV in liquid for pigs via the oral route was 10^1.0^ TCID_50_^24^ and The successful transmission of ASFV via shared needles from the ASF-H and ASF-M subgroups was expected and adds to the knowledge on internal biosecurity measures in swine herds: in a context where ASFV is not eradicated, disposable needles should be considered as a single-use material. While this recommendation may be complied with on experimental farms, it is not realistic for conventional pig farms where parenteral vaccination of large number of pigs by intramuscular injections is a routine^10^. The results obtained with the ID needle-free vaccination device in the present study are in favor of recommending that conventional farms to switch to ID needle-free vaccination whenever it is possible. This type of vaccination has been demonstrated as an economically viable option on large farrow-to-finishing farms, where it enhances both human well-being and system performance^25^.

Needle-stick injury is well-known as a mode of accidental hematogenous transmission of pathogens in humans and is preventable. It is estimated that some 2 million cases of such injuries occur yearly worldwide among health-care workers^26^, even though the single use of disposable needles is strictly applied, and prevention is efficient at reducing accident prevalence. No such statistics are available from the animal health sector, but such accidents do occur. Switching to ID needle-free vaccination might also improve safety to stockmen and other animal health professionals.

One of the limitations of our study was the limited number of pigs included in each seeder subgroup. We adhered to the Replace, Reduce and Refine (3Rs) policy in animal studies to minimize animals infected or euthanized. Six pigs per group reached statistical power and were adequate to demonstrate transmission. Another limitation was the usage of Ct values as a proxy to infectious virus titers. Transmissibility has however been obtained with seeder pigs that had very limited genomic loads at the moment of needle contamination (D7 PC). This reinforces the reliability of using Ct as an indicator for viremia. Lastly, it is impossible to determine the origin of the infection of pen-mate sentinel pigs once the first case broke in a room (injection or contact). However, the fact that the negative control group remained disease and viremia-free over the 42 days of the experiment points to the relevance of considering that the first pig in each sentinel room that broke with ASF had been infected by the contaminated needle, and not by on-site cross-contamination, hence demonstrating needle transmission.

Taken together, these data, along with those recently published on PRRSV hematogenous transmission via needles, should weigh in favor of considering a wider usage of needle-free devices and ID vaccination in the Asian swine industry. This would be part of the modernization of both disease control strategies and production systems, which are considered highly desirable.

## Supplementary Information

Supplementary Table 1.

## Data Availability

The datasets generated and/or analyzed during the current study are available from the corresponding author.
