# Supplementary material for: Intradermal needle-free injection prevents African Swine Fever transmission, while intramuscular needle injection does not
Source: Sci Rep. 2023 Mar 21;13:4600. doi: 10.1038/s41598-023-31199-2 (PMC10028754; doi:10.1038/s41598-023-31199-2)
Supplement: Supplementary file 1 — Supplementary Table 1. [file 41598_2023_31199_MOESM1_ESM.docx]

**Table 1 supplementary material**

Quantitative PCR Ct values of seeder pigs following ASFV challenge (challenge performed at D0; D = sudden death or euthanasia).

| Subgroup |  | Pig no. | D0 | D3 | D5 | D7 | D10 | D14 | D21 | D28 | D35 |
| --- | --- | --- | --- | --- | --- | --- | --- | --- | --- | --- | --- |
| ASF-H |  | SE_1-1 |  | 34.90 | 35.20 | 27.70 | D | D | D | D | D |
|  |  | SE_1-2 |  | 31.40 | 18.80 | 14.70 | 38.90 | 16.90 | D | D | D |
|  |  | SE_1-3 |  | 36.60 | 30.40 | 34.10 | 37.50 | 18.90 | 16.90 | D | D |
|  | AVG Ct |  | 35.00 | 34.30 | 28.13 | 25.50 | 20.55 | 17.90 | 16.90 |  |  |
|  | SD Ct |  | 0.00 | 1.93 | 6.22 | 7.20 | 1.65 | 1.00 | 0.00 |  |  |
|  | No. of pigs with Ct<35 |  | 0/3 | 0/3 | 2/3 | 2/3 | 2/2 | 2/2 | 1/1 |  |  |
|  |  |  |  |  |  |  |  |  |  |  |  |
|  | No. of deaths/total no. pigs |  | 0/3 | 0/3 | 0/3 | 1/3 | 1/3 | 1/3 | 2/3 | 3/3 | 3/3 |
| ASF-M |  | SE_2-1 |  | 35.90 | 37.90 | 32.30 | 32.30 | D | D | D | D |
|  |  | SE_2-2 |  | 34.60 | 28.60 | 31.60 | 27.60 | 34.80 | D | D | D |
|  |  | SE_2-3 |  | 32.90 | 32.20 | 31.70 | 38.00 | 38.80 | 17.00 | D | D |
|  | AVG Ct |  | 35.00 | 34.47 | 32.90 | 31.87 | 30.50 | 20.55 | 17.00 |  |  |
|  | SD Ct |  | 0.00 | 1.04 | 3.33 | 0.29 | 1.93 | 1.65 | 0.00 |  |  |
|  | No. of pigs with Ct<35 |  | 0/3 | 0/3 | 2/3 | 2/3 | 2/3 | 2/2 | 1/1 |  |  |
|  |  |  |  |  |  |  |  |  |  |  |  |
|  | No. of deaths/total no. pigs |  | 0/3 | 0/3 | 0/3 | 0/3 | 0/3 | 1/3 | 2/3 | 3/3 | 3/3 |
| ASF-L |  | SE_3-1 |  | 34.40 | 34.30 | 36.40 | 32.20 | 36.80 | 32.30 | 37.60 | D |
|  |  | SE_3-2 |  | 36.80 | 37.50 | 37.50 | 39.40 | 37.50 | 32.50 | 36.60 | 22.60 |
|  |  | SE_3-3 |  | 37.50 | 37.50 | 34.50 | 34.30 | 26.30 | 35.70 | 36.70 | 20.00 |
|  | AVG Ct |  | 35.00 | 36.23 | 36.43 | 36.13 | 35.30 | 36.67 | 36.43 | 35.90 | 22.67 |
|  | SD Ct |  | 0.00 | 1.22 | 1.42 | 1.09 | 2.73 | 0.64 | 0.49 | 1.00 | 1.82 |
|  | No. of pigs with Ct<35 |  | 0/3 | 0/3 | 0/3 | 0/3 | 0/3 | 0/3 | 0/3 | 1/3 | 3/3 |
|  |  |  |  |  |  |  |  |  |  |  |  |
|  | No. of deaths/total no. pigs |  | 0/3 | 0/3 | 0/3 | 0/3 | 0/3 | 0/3 | 0/3 | 0/3 | 0/3 |
